# Direct Current Stimulation in Cell Culture Systems and Brain Slices—New Approaches for Mechanistic Evaluation of Neuronal Plasticity and Neuromodulation: State of the Art

**DOI:** 10.3390/cells10123583

**Published:** 2021-12-19

**Authors:** Nadine Euskirchen, Michael A. Nitsche, Christoph van Thriel

**Affiliations:** IfADo—Leibniz Research Center for Working Environment and Human Factors, 44139 Dortmund, Germany; Nitsche@ifado.de (M.A.N.); Thriel@ifado.de (C.v.T.)

**Keywords:** DCS, cell culture (in vitro), brain slices (ex vitro), neuroplasticity, neuromodulation

## Abstract

Non-invasive direct current stimulation (DCS) of the human brain induces neuronal plasticity and alters plasticity-related cognition and behavior. Numerous basic animal research studies focusing on molecular and cellular targets of DCS have been published. In vivo, ex vivo, and in vitro models enhanced knowledge about mechanistic foundations of DCS effects. Our review identified 451 papers using a PRISMA-based search strategy. Only a minority of these papers used cell culture or brain slice experiments with DCS paradigms comparable to those applied in humans. Most of the studies were performed in brain slices (9 papers), whereas cell culture experiments (2 papers) were only rarely conducted. These ex vivo and in vitro approaches underline the importance of cell and electric field orientation, cell morphology, cell location within populations, stimulation duration (acute, prolonged, chronic), and molecular changes, such as Ca2+-dependent intracellular signaling pathways, for the effects of DC stimulation. The reviewed studies help to clarify and confirm basic mechanisms of this intervention. However, the potential of in vitro studies has not been fully exploited and a more systematic combination of rodent models, ex vivo, and cellular approaches might provide a better insight into the neurophysiological changes caused by tDCS.

## 1. Introduction

The targeted modulation of brain activity via controlled magnetic or electric stimulation is a valuable research tool in neuroscience, as well as an emerging clinical intervention in neurological and psychiatric diseases [1].

Within the group of non-invasive brain stimulation techniques (NIBS), tDCS has developed into a frequently used approach in neuroscience. tDCS is applied with surface electrodes and with low intensity electrical currents [2]. Dependent on the stimulation protocol, the intervention induces acute, but also prolonged, shifts of cortical excitability [3,4,5].

Effects of tDCS are supposed to be accomplished by a subthreshold modulation of neuronal membrane resting potentials that alters cortical excitability, and can subsequently induce more enduring effects related to neuroplasticity [3]. With respect to the after-effects of this intervention, tDCS over the motor cortex of healthy humans alters cortical excitability for a couple of minutes up to hours [4,6] after intervention. Furthermore, it was shown that tDCS effects depend on the current flow direction in relation to the targeted neuron populations or brain regions [3,7,8]. For the primary motor cortex model in humans, stimulation with the anode placed over the motor cortex target and a cathodal return electrode positioned over the contralateral supraorbital area increased motor evoked potentials (MEPs) (long-term potentiation (LTP)-like mechanism) and reversed electrode positioning reduced MEPs (long-term depression (LTD)-like mechanism) [3,4,5]. However, with other return electrode positions, no clear physiological effects emerged [3].

A considerable number of studies in humans have explored the physiological foundation of the effects of tDCS. Pharmacological studies have shown that the acute effects of short-lasting tDCS are not synaptically driven, but depend on membrane polarization, because the effects of depolarizing anodal tDCS were prevented by voltage-gated ion channel blockers, but not by glutamatergic N-methyl-D-aspartate (NMDA) receptor block [9]. In contrast, neuroplastic after-effects were prevented by NMDA receptor block, but increased by NMDA receptor enhancement [9,10]. This suggests that plasticity induced by tDCS depends on the glutamatergic system and involves calcium-dependent mechanisms. Moreover, Tropomyosin receptor kinase B (TrkB), the main receptor of brain derived neurotrophic factor (BDNF), was found to be involved in LPT-like effects of DCS [7]. Furthermore, tDCS reduced γ-amino-butyric acid (GABA) activity independent from stimulation polarity [11], which might have a gating effect on tDCS-induced plasticity. Early in vivo animal studies moreover suggest that after-effects of DCS, which last for more than 3 h and thus resemble late phase plasticity, require protein synthesis [12]. Beyond these regional effects of tDCS, it was shown that this intervention has an impact on cortical networks in terms of functional connectivity alterations and topological functional organization [13].

Based on respective physiological effects, tDCS has a relevant impact on psychological processes including perception, executive functions, and learning and memory, amongst others [14]. Some heterogeneities of these effects have been described between studies which underline the importance of improving mechanistic understanding of this intervention to optimize effects.

In the clinical domain, tDCS emerged as a valuable non-invasive brain stimulation tool to ameliorate symptoms in diseases accompanied by pathological alterations of cortical activity and plasticity such as depression, schizophrenia, pain syndromes, epilepsy, and in rehabilitation, amongst others [15,16]. However, heterogeneities of results are observed between studies, which might be caused at least partially by intervention protocol differences, and thus stress the need to understand basic tDCS effects better to improve the efficacy of interventions.

Although various mechanistic studies are available in humans [17,18,19] and also in animal models [7,20,21], the exact molecular mechanisms underlying the neuromodulatory effects of tDCS are yet not fully understood. Therefore, gathering more direct evidence using sophisticated neurobiological techniques such as cell-based assays (in vitro), brain slices (ex vivo), or in vivo animal models are required to supplement existing knowledge. Pelletier and Cicchetti summarized relevant research in 2015 [22]. They described cellular effects related to electrotaxis, metabolism, differentiation, cell orientation, as well as galvanotropism [23,24,25,26,27,28,29,30,31,32,33,34,35,36], to name a few. However, all these morphological and structural changes have only been observed after either long-term stimulation, e.g., for 12 h [37], or with high electric field intensities of, e.g., 55.5 A/m^2^ [38], which differ from those applied in healthy humans and patients. In human clinical studies, stimulation duration is typically around 20 min and electrical field strength is ≤1 V/m with a maximum current density of about 0.28 A/m^2^ [39,40]. Nevertheless, Pelletier and Cicchetti also summarized animal data investigating DCS protocols more closely comparable to those applied in humans. These showed effects on brain physiology by different mechanisms, including the dependency on direction and impact of DC-induced membrane polarization on the relative orientation of the cells within the electric field, the involvement of modulation of presynaptic compartments, and modulation of action potential generation in efferent neurons [8,41]. Beyond these neuronal effects, DC stimulation has an impact on various other physiological processes.

DC electric fields can influence inflammatory processes (both anti- and pro-inflammatory [42]), structural neuroplasticity (neurite outgrowth [43]), neurogenesis (in case of directed neural stem cell migration towards a lesion or damaged location [30]), and angiogenesis [44].

To sum up, based on available knowledge, it is suggested that:

- the primary effects of DC stimulation are non-synaptic membrane polarization effects which are dependent on cell and cell-compartment orientation relative to the induced electric fields;
- secondary neuronal effects of DC stimulation on plasticity require initial polarization, spontaneous neuronal activity, and are driven by glutamate, gated by GABA activity reduction, and require BDNF expression;
- long-lasting effects resembling time windows of late phase plasticity require protein synthesis;
- stimulation dosages in animal and cellular studies are often relevantly higher than those used for interventions in humans; and
- tDCS has also non-neuronal effects.

Available knowledge about the precise mechanisms underlying tDCS, immediate and after-effects, is however still incomplete and largely derived from indirect approaches in humans and animals. To shed light on recent progress in the field, this review aims to provide an overview about mechanistic in vitro and ex vivo studies investigating DCS effects on neurons and neuronal populations. To this aim, we will shortly summarize selected parts of the review by Pelletier and Cicchetti from 2015 regarding the current state of knowledge about cellular and molecular mechanisms of the effects of DC electric field stimulation. Moreover, we will put together new information obtained from 2015 up to now regarding brain slice and cellular system approaches in greater detail.

## 2. Materials and Methods

### 2.1. Literature Search

**Figure 1:**
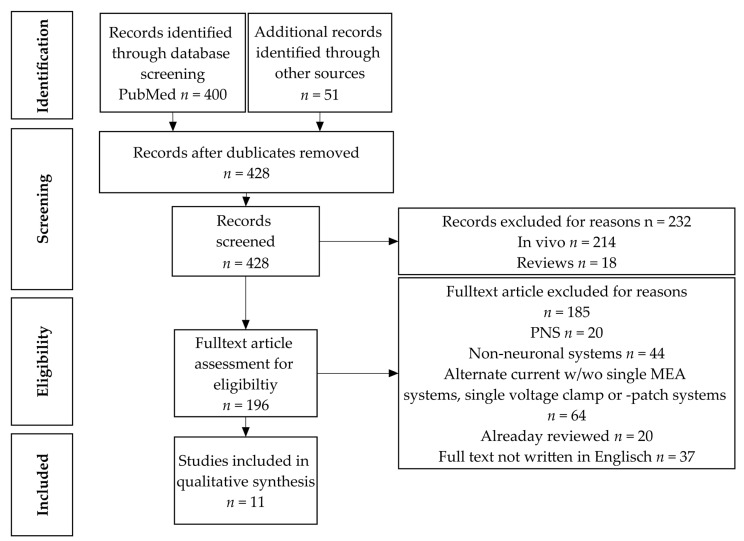
Schematic overview of the literature evaluation and reduction. The diagram shows the four steps of the procedure to identify contributions suited for the present review, starting on the top with the whole literature search obtaining 451 papers, progressing to the screening step in the middle leading to 428 contributions. A total of 196 articles were assessed for eligibility and 11 publications were finally identified as eligible and included. W/wo: with/without; PNS: peripheral nervous system; MEA: multi-electrode array.

This systematic review was conducted in accordance with the 2020 PRISMA guidelines [45]. A comprehensive systematic search of articles was executed in PubMed using the terms “electrical stimulation”, electri*, neuro*, “in vitro”, plasti*, “direct current stimulation” not transcranial, not “in vivo”, micropolarization, micro-polarization, “micro polarization”, “anodal polarization”, “cathodal polarization”, “galvanic electrical stimulation”, “dominant focus”, LTP, and LTD. Articles published until 2021 were selected, and the last update was performed in June 2021. This search strategy yielded 400 articles that were further evaluated. A further 51 articles were identified by a literature search using Google Scholar and screening of already available reviews (see Figure 1).

### 2.2. Inclusion Criteria, Literature Screening, and Eligibility

After screening title, abstract, and in cases when the obtained information was not sufficient to make conclusions, the material and methods sections, duplicates (*n* = 23), and all in vivo papers (*n* = 214) as well as reviews (*n* = 18) were excluded. The remaining papers were subdivided into two groups: in vitro (cell culture) and ex vivo (slices). Further exclusion criteria were non-neuronal cells/systems (*n* = 44) and peripheral nervous system (PNS) preparations—such as whole nerve extractions (*n* = 20). Furthermore, all non-DC stimulations (alternating current (AC), multi-electrode array (MEA) systems, single cell patch- or voltage-clamp measures; *n* = 64) were excluded, too. Articles already reviewed by McCaig et al. (2005) and Pelletier and Cicchetti (2015) [35,46] (*n* = 20) and finally all papers whose full text was not written in English (*n* = 37) were excluded. For an overview, the procedure and results of the literature screening, eligibility evaluation, and inclusion are given in Figure 1.

## 3. Results

Two DC papers investigating neuronal cell systems and nine papers using brain slice systems were identified which fulfilled all requirements (see Table 1). We grouped these scientific contributions into three sections: 1. Neuronal orientation/morphology/alignment/location, 2. stimulation duration, and 3. molecular changes.

### 3.1. Orientation of the Electric Field in Relation to Neuronal Population Morphology/Alignment, and Location

Kronberg et al. (2017) used rat hippocampal slices and applied current strengths of 100–200 µA for 45 s, and 3, 15, and 30 min. The electric field was oriented parallel to the somato-dendritic axis of cornu ammonis (CA1) pyramidal neurons. Trains of 900 electrical pulses at varying frequencies (0.5, 1, 5, and 20 Hz) were applied to generate plasticity before DCS, and field excitatory postsynaptic potentials (fEPSPs) were monitored at the dendritic level. Cathodal DCS enhanced LTP in apical dendrites and anodal DCS enhanced LTP in basal dendrites. Interestingly, both anodal and cathodal DCS reduced LTD in apical dendrites and DCS had no effect on weakly active synapses during NMDA block [47]. In a subsequent study, the same group showed that anodal DCS applied during theta burst stimulation (TBS) for some seconds enhanced Hebbian LTP [21].

Rahman et al. applied 10–150 µA currents for 3–5 s in rat motor cortex slices. Electrical fields were oriented orthodromic to L2/3 to induce fEPSP alterations. The postsynaptic membrane polarization during DCS and ongoing presynaptic activity induced by a train of presynaptic inputs delivered with constant or Poisson-distributed stimuli resulted in sustained and cumulative enhancement of fEPSPs [48].

Chakraborty et al. (2018) stimulated mouse coronal prefrontal cortical slices for 1 min with current strengths between 34.8 and 58.3 µA parallel and orthogonal to the dendrito-axonic axis of layer-V pyramidal neurons. They used recordings of membrane polarization (V/m) as readout. Chakraborty et al. (2018) showed that suprathreshold stimulation (important for, e.g., deep brain stimulation) induces action potentials at axon terminals, whereas subthreshold stimulation (important for DCS) modulates synaptic efficacy through axon terminal polarization. Moreover, only orientation of the electrical field parallel to the dendrite-axonal axis of the neurons induced these effects [49].

Recent experiments added the feature of cell localization as an important aspect affecting DCS effects. In contrast to previous studies, they used mouse and human cortical slices stimulated with 400 µA for 25 min. Slices were oriented orthogonal to the pia and parallel to the vertical inter-layer primary motor cortex (M1) projections with the cathode proximal to the cortical pia surface and the anode beneath the subcortical white matter. Their experiments focused on cortical excitability in human and mouse slices with cathodal stimulation. DCS generated LTD in superficial cortical layers, and LTP-like plasticity in deep cortical layers [52].

### 3.2. Acute, Prolonged, and Chronic DCS

Beyond application of DCS for some seconds, which has been discussed above and induces acute effects on neuronal membranes but no plasticity, DCS can also be applied for prolonged (minutes, effect on plasticity), or even chronic time courses (hours to days, effects on cell migration and neuronal orientation) [22].

Some articles already discussed in the previous section applied prolonged stimulation, such as Kronberg et al. (2017) and Sun et al. (2020) [47,52], and other contributions with prolonged stimulation protocols will be discussed in the next section.

Reato et al. (2015) for instance focused on the after-effects of prolonged DCS on gamma power and multi-unit activity (MUA). They used rat hippocampal slices and stimulated these for 10 min at a field strength varying from −20 up to + 20 mV/mm parallel to CA3 pyramidal neurons after induction of gamma oscillations by carbachol. They defined positive fields as anodal (0 to 20 mV/mm) and negative fields as cathodal (−20 to 0 mV/mm). Electrodes were oriented parallel to CA3 pyramidal cells. Their results showed altered gamma power and multi-unit activity (MUA) in a polarity-specific manner after 10 min of DCS: −20 and −10 mV/mm led to an acute downregulation of MUA and gamma power while +10 and + 20 mV/mm upregulated both. These results persisted for 10 min after stimulation [53]. Latchoumane et al. (2018) applied repeated prolonged DCS on embryonic stem cell (ESC)-derived neurons and glial cells after L-Glutamate-induced impairment of neuronal maturation. They treated mouse ESCs with currents of 10 µA over 5 days, 15 min per day. The intervention enhanced neuronal excitability as well as network synchronization. They furthermore demonstrated an upregulation of the NMDA receptor subunit NR2A, and Ras-related protein RAB3A in mouse Hb9 ESC-derived neurons and glial cells [50].

Zhao et al. (2015) applied chronic DCS in a stem cell model. They stimulated mouse neuronal precursor cells (NPCs) for 90 min with a current strength of 0.25 nA. The DC electric field enhanced mobility and caused cultured NPC migration to the cathode. A calcium-dependent mechanism was explored by adding the calcium chelator Ethylene glycol-bis(2-aminoethylether)-N,N,N’,N’-tetraacetic acid (EGTA) into the medium during DCS. The results showed that EGTA significantly decreased cell migration during DCS [54].

### 3.3. Molecular Changes—Plasticity and Neuromodulation

It has been suggested that the after-effects of tDCS are related to molecular mechanisms which play a vital role in activity-dependent synaptic plasticity. The molecular changes after DCS regarding plasticity and neuromodulation have been explored in the following contributions.

Ranieri et al. (2012) applied DCS with 200–250 µA for 20 min in rat hippocampal slices, with the electrical field oriented parallel to the somato-dendritic axis of CA1 pyramidal cells. They demonstrated a modulatory effect of DCS on LTP induced by a standard high frequency stimulation (HFS) protocol consisting of four trains of 50 stimuli at 100 Hz (500 ms each) repeated every 20 s [55]. Specifically, anodal DCS increased, while cathodal DCS decreased LTP at CA3-CA1 synapses in rat hippocampus. Furthermore, an induction of early genes such as c-fos and Zif268, which are relevant for structural plasticity [56], was observed following DCS.

Chang et al. (2015) stimulated mouse thalamocingulate brain slices with the electrical field oriented parallel as well as perpendicular to the direction of axodendritic fibers to investigate suppressive effects of DCS on the anterior cingulate cortex (ACC). DCS for 15 min at 400 µA induced LTD. Excitatory postsynaptic currents (EPSCs) were monitored via MEA and patch clamp recordings. DCS significantly decreased epileptic EPSCs, which were generated by 4-aminopyridine treatment prior to DCS. Furthermore, the NMDA receptor antagonist D-1-2-amino-5-phosphonopentanoic acid (APV) totally abolished this DCS effect [20].

Cortical excitability alterations due to cathodal DCS have also been explored in another study. mGluR5-mTOR signaling was identified as a novel pathway by which tDCS modulates cortical excitability. Mouse and human coronal slices oriented parallel or orthogonal to M1 fibers (layer V to II/III projections) were exposed for 10 or 20 min to current strengths of 300 or 400 µA. DCS induced LTD in both human and mouse cortices. These effects were abolished by an mGluR5-negative allosteric modulator but stabilized by a mGluR5-positive allosteric modulator [18].

## 4. Discussion

Stimulation with weak direct currents as a tool to affect brain excitability and neuroplasticity was so far primarily used in vivo in humans and animal models, including basic and clinical applications. Mechanistic knowledge is not only relevant with respect to basic science studies, but also for application of DCS in clinical domains, to better understand its impact and optimize effects. At the mesoscale level, respective knowledge was obtained in early in vivo human and animal studies, which showed relatively homogeneous effects on cortical excitability, and also revealed some mechanisms at the level of receptor and transmitter contribution [40,57]. These studies are however not well suited to explore detailed mechanisms at the cellular and molecular levels and pharmacological studies used for exploration of mechanisms provide only indirect evidence. To explore the mechanisms of action of this intervention in detail, ex vivo and in vitro DCS studies are important to unravel the mechanistic foundations of the impact of tDCS on cells, cell compartments, and molecular physiology. Respective studies reviewed by Pelletier and Cicchetti in 2015 and in the present review show more complex and specific results compared to those obtainable at the mesoscale level, and thus advance knowledge about DCS effects relevantly.

### 4.1. Localization

In vivo effects of tDCS at the mesoscale level, obtained by methods which monitor excitability by field potential recordings in animal models or evoked potentials in humans showed that with conventional stimulation protocols stimulation with the anode over the target area enhances cortical excitability, while cathodal stimulation reduces it [40,58]. These studies are however not suited to reveal the detailed effects of stimulation at the single neuron level.

With respect to the effects of DCS on different cortical layers, modeling results suggested layer-specific effects of DCS at the whole brain level [59], and were furthermore experimentally confirmed by ex vivo studies. Sun and co-workers (2020) showed that the cortical layer has major effects on the directionality of DCS effects [52], which might be due to layer-specific electric field to neuronal orientation alignment, but also to layer-specific differences of electrical field strength. DCS effects in slice preparations thus show a higher heterogeneity than results at the mesoscale level and might reveal reasons for heterogeneity of effects due to different stimulation intensities, and electrical field orientations. At the whole neuron level, Radman et al. (2009) showed moreover that the cellular morphology of cortical neurons affected DCS effects in different layers of the brain. Pyramidal neurons were more sensitive to polarization than other neurons. Moreover, layer V/VI pyramidal neurons were more sensitive to stimulation than layer II/III pyramidal neurons [60].

Beyond layer specificities, intracellular compartment-specific effects of DCS have also been described. Kronberg et al. (2017) monitored fEPSPs in apical and basal dendrites, and found discernible modulatory effects of anodal and cathodal stimulation on LTP in apical and basal dendrites, whereas both stimulation polarities reduced dendritic LTD [47]. With respect to the contribution of the soma, and axon to DCS effects, Chakraborty et al. (2019) described a modulatory effect of DCS on synaptic and electric activity of axons, whereas the effect on the neuronal soma was relatively minor [49]. These studies enhance information about layer-specific effects of DCS, as well as the contribution of different neuronal compartments, which might help to explain heterogeneities of DCS effects to a larger degree, and to guide electrode placement and stimulation dosages to target specific neuronal phenotypes in different cortical layers.

### 4.2. Physiological Mechanisms

Results from in vivo experiments in humans suggest that the acute effects of DCS are driven by membrane potential changes and not by alteration of synaptic efficacy [9]. Recent studies confirm those findings in ex vivo experiments by describing a de- and hyperpolarizing effect of acute DCS on axons [48,49] and dendrites [47].

In addition to acute polarization effects, which emerge immediately, stimulation for a few minutes results in neuroplastic after-effects, which depend on glutamatergic mechanisms including NMDA but also AMPA (α-amino-3-hydroxy-5-methyl-4-isoxazolepropionic acid) receptors, as suggested by in vivo experiments in humans and animal models [9,19]. Ex vivo studies have shown similar plasticity-inducing effects of DCS [7,18,52].

Beyond these direct plasticity-inducing effects, tDCS also alters plasticity induced by other intervention protocols. This was rarely explored directly in vivo [61], but might be an important foundation for its impact on learning and memory formation [62]. In accordance, recent ex vivo studies showed that DCS has modulatory effects on LTP as well as on LTD [21,47,51].

Taken together, the ex vivo studies reviewed here confirm polarization of neuronal membranes as a primary mechanism of DCS, but also the induction of neuroplasticity. Moreover, these studies confirm a modulatory effect of DCS on plasticity induced by other interventions.

### 4.3. Molecular Mechanisms

A couple of ex vivo studies have been conducted to explore the molecular mechanisms underlying neuroplastic effects of DCS. Based on animal and human in vivo studies, these are suggested to share basic mechanisms of LTP and LTD, including involvement of glutamatergic receptors, including NMDA receptor and calcium dependency [9,63,64]. These suggestions were confirmed by ex vivo studies. Chang and co-workers showed an involvement of NMDA receptors in DCS-induced LTD in ex vivo [20], and comparable results were also described in other studies [47,52]. Sun et al. (2020) moreover identified a contribution of metabotropic glutamate receptors as an additional glutamatergic pathway involved in DCS-induced LTD [18]. Furthermore, mTor signaling was found as a novel (interaction) pathway affected by DCS. This shows that not only ionotropic glutamate receptors, but also metabotropic receptors related to intracellular signaling pathways (e.g., kinase-dependent pathways) are involved in DCS effects [52].

Additional evidence for synaptic plasticity mechanisms induced by chronic DCS was obtained by an in vitro approach with neuronal cell cultures. Upregulation of the NMDA receptor subunit NR2A, BDNF, as well as RAB3A in mouse Hb9 ESC-derived neuron and glial cells due to repeated prolonged anodal stimulation was observed [52]. Ranieri et al. (2012) extended knowledge about modulatory DCS effects on plasticity further by exploring the contribution of genes, molecules, and enzymes. They observed an induction of structural plasticity-relevant genes, such as c-fos and Zif268 after DCS [51]. This confirms and extends early work which showed a dependency of late-phase plasticity induced by tDCS on protein synthesis [12].

Cell migration caused by chronic DCS has been described to some extent before. Zhao et al. (2015) expanded mechanistic knowledge about these effects to the molecular level by describing calcium-dependent migration of the cells to the direction of the cathode as an underlying mechanism [54].

Taken together, the available ex vivo studies in which molecular mechanisms of DCS were explored not only confirmed the dependency of respective effects from the glutamatergic system, but also identified additional glutamatergic receptors involved, as well as early genes, enzymes, and mechanistic details of cell migration induced by chronic DCS.

## 5. Conclusions and Outlook

Since the reintroduction of tDCS as a non-invasive brain stimulation technique about 20 years ago, numerous studies have been conducted to explore mechanisms of action of this intervention in vivo. So far, many of these studies have been carried out in humans.

Regarding the discrepancy in the available literature, it must be kept in mind that sometimes clinical applications are under strong pressure being applied without exploring and knowing the underlying mechanisms conclusively. This is also true for other brain stimulation techniques such as transcranial magnetic stimulation (TMS) or focused ultrasound stimulation (FUS), but applies also to other interventions. As pointed out by Müller-Dahlhaus and Vlachos (2013), and Tang et al. (2017), a literature screening for the TMS techniques (single trial or repetitive) also revealed more clinical and human trials than published ex vivo or in vitro research [65,66]. Recently, the benefits of in vitro research were shown nicely in the investigation of the involvement of glia cells in TMS [67]. This contribution showed elegantly how in vivo and in vitro research can be combined in the context of invasive and non-invasive brain stimulation techniques. Together with the results of our review, this shows that there is development in this field. However, translation from in vitro studies to in vivo humans is not trivial, including a paucity of established and widely accepted animal models of brain stimulation, mainly due to various anatomical differences and the lack of comparable stimulation devices for the usual pre-clinical cellular and animal models [65,66].

Animal and cellular studies are however required since the opportunities to identify mechanisms of action at the cellular and molecular levels are limited with respect to studies in humans. This review reveals detailed knowledge about the location, physiology, and molecular mechanisms of DCS obtained by ex vivo studies, but also the potential of well-designed in vitro systems that have not been fully exploited. However, many promising attempts were made within the last 5–10 years in DCS animal models, and these might have implications not only for basic, but also for applied research.

The findings of the reviewed papers indicate a complex mechanism of DCS effects. This includes determination of the effects by protocol specifics such as stimulation duration, cell location in relation to the electrode and within a neuronal population, as well as orientation of the cells relative to the electric field. Moreover, effects in a given neuron are not uniform but compartment-specific (axons/soma/dendrites). Furthermore, effects between different brain layers are not uniform, which might have anatomical or methodological reasons.

In vivo human as well as animal trials are limited with respect to identification of mechanistic details of the effects of DCS. Moreover, translation from rodent brain models to humans must be considered critically as the morphology is rather different between these species. Therefore, primate models would be ideal animal models [68]. However, like in human studies, primate models are subjected to high ethical constrains including the avoidance of highly invasive methods. The few available publications with macaques indicate that tDCS affects brain connectivity (see [68]) and is therefore able to further explore the physiological effects that might underlie effects in humans such as the synchronization of theta oscillations in patients with schizophrenia [69]. Accordingly, in vivo experiments with primates are promising models for such complex tDCS interventions. Moreover, the optimization of current flow and its modeling might further benefit from non-human primates in vivo experiments using implanted multi-site intracranial Utah arrays [70]. Here, Alekseichuk et al. (2019) provided a comprehensive comparison of computational models for transcranial magnetic and electric stimulation in mouse, monkey, and human [71]. As discussed for the hiPSC-derived neuronal in vitro systems, future mechanistic DCS research should consider the inclusion of primate models where appropriate.

Nevertheless, as shown in our results, ex vivo and in vitro assays are essential as effects of acute, prolonged, and even chronic DCS can be assessed in these assays. It should however be mentioned that these are complementary to in vivo studies. Cell cultures and brain slices are advantageous when conducting mechanistic studies, while, e.g., behavioral effects and the effects of DCS at the systems level can only be addressed in vivo. One example of the adjunctive value of both in vivo and in vitro studies is the identification of modulatory effects of DCS on LTP in vitro [21,47,51], which explains the in vivo data that tDCS improves learning and memory formation [62,72].

However, evidence from ex vivo studies [7] supports the concept that DCS can improve learning and memory formation when it is connected to learning conditions, as shown in several in vivo publications [73,74,75]. Because of differences of the physiological and chemical milieu of in vivo and ex vivo studies, ideally the results obtained in vitro should be confirmed by in vivo studies. Ex vivo and in vitro studies can provide very specific information about the molecular changes induced by DCS due to the much larger repertoire of pharmacological or even genetic manipulations, as compared to in vivo studies including those in humans. With respect to in vitro studies embryonic, neuronal stem cell derived neurons, as well as hiPSC are increasingly used as an alternative to primary neurons from rodents. However, embryonic and neuronal stem cell-derived neurons are ethically debated and furthermore have the disadvantage of extensive cell culture prior use [76]. In seizure liability assessment research, hiPSC-derived neuronal models have already been introduced as alternative in vitro models for drug screening [77]. In combination with non-invasive recording of drug-induced alterations of spontaneous activity using MEAs, this recent review concludes that further validation and standardization is needed to mimic the human in vivo situation. Thus, in the context of in vitro research trying to unravel the molecular mechanisms of tDCS on a cellular level, these developments should be integrated. As pointed out in our review, DCS effects strongly depend on the current flow through dendrites and axons that must be organized in a way representing the in vivo organization of certain brain structures such as the hippocampus. Currently, rodent brain slices are the only ex vivo approach that fulfils this prerequisite as hiPSC-derived neuronal models involving 3D scaffolds are currently in a very early phase of development [78]. Thus, future in vitro studies investigating the effect of DCS on cellular processes related to neuronal plasticity might consider the use of hiPSC-derived neuronal models.

Nevertheless, the pipeline of in vivo–ex vivo–in vitro studies is crucial for the overall view as every system has its strengths and weaknesses and is required to answer specific questions. Transferability from one system to another is critical, as the different systems need to be calibrated to their specific setups (in vivo, ex vivo, in vitro) and settings (stimulation duration, intensity, etc.).

Taken together, experiments in cells, slices, and in vivo animal models help to unravel the pathways, molecules, neurotransmitter systems, and genes involved in DCS effects. Transfer and adaptation of these settings and results to the human in vivo situation might be principally possible, but some limitations, related to the level of spontaneous activity in the various systems, the chemical milieu, and others should be kept in mind. Nevertheless, experimental cascades from in vivo to ex vivo and in vitro approaches have the potential to unravel the detailed mechanistic effects of DCS in the future.

## Figures and Tables

**Table 1:** Summary of DCS studies conducted in animal and human brain cells and brain slices. 4-AP: 4-aminopyridine; ACC: anterior cingulate cortex; CA1: cornu ammonis 1; d: diameter; DCS: direct current stimulation; ESC: embryonic stem cell; EPSC: excitatory postsynaptic currents; fEPSP: field excitatory postsynaptic potential; KA: kainic acid; l: length; LII/III, V: Layer 2/3, 5; LTP: long-term potentiation; LTD: long-term depression; MUA: multiunit activity; NMDA: N-methyl-D-aspartate; DBS: deep brain stimulation; N/A: not available; MEA: multi-electrode array; mGluR5-mTOR: metabotropic glutamate receptor subunit 5—mechanistic target of rapamycin; NPCs: neural precursor cells; RAB3A: Ras-related protein; TBS: theta burst stimulation; qRT-PCR: quantitative real-time polymerase chain reaction.

Reference	Origin	Tissue	Stimulation Duration (min)	Field Strength (mV/mm)	Electrode Size (mm)	Current Strength (µA)	Slice Orientation	Readout	Results/Observations
Kronberg et al., 2017 [47]	Rat	Hippocampal slices	0.75, 3, 15 and 30	20	1 d, 12 l	100–200	Parallel to somato-dendritic axis of CA1 pyramidal neurons	Recording of fEPSP before and after plasticity induction with low and high frequency suprathreshold electrical stimuli combined with DCS	Cathodal DCS enhances LTP in apical dendrites; anodal enhances LTP in basal dendrites; both reduce LTD in apical dendrites; no effect in weakly active synapses or during NMDA receptor block
Rahman et al., 2017 [48]	Rat	Motor cortex slices	0.05–0.08	10–20	N/A	10–150	Orthodromic stimulation of LII/III	Recording of fEPSPs	Presynaptic inputs were delivered with constant or Poisson-distributed stimuli prior to single DCS stimuli; postsynaptic voltage response during DCS and ongoing presynaptic activity results in sustained and cumulative changes in fEPSP; regulated by synaptic efficacy, number of active inputs, and rate of presynaptic activity
Chakraborty et al., 2018 [49]	Mouse	Coronal pre- frontal cortical slices	1	5	N/A	58.3–34.8	Parallel and orthogonal to dendrito-axonic axis of L-V pyramidal cells	Recording of membrane polarization per V/m of effective electric field	Suprathreshold stimulation (important for e.g., DBS) induces action potentials at terminals; subthreshold stimulation (important for DCS) modulates synaptic efficacy of axon terminal polarization; significant effect after parallel-, no effect after orthogonal orientated polarization
Kronberg et al., 2020 [21]	Rat	Hippocampal slices	0.06	20	1 d, 12 l	100–200	Parallel to somato-dendritic axis of CA1 pyramidal neurons	Recording of fEPSPs	Anodal DCS boosts LTP of Hebbian plasticity-dependent pathways during the induction of LTP with TBS
Latchoumane et al., 2018 [50]	Mouse	ESC-derived neuron and glial cells	15, (5 days) day 14 cathodal, day 15–19 anodal	N/A	N/A	10	/	qRT-PCR analysis after chronic DCS on ESC-derived neurons after L-Glutamate administration	Upregulation of NMDA receptor subunit NR2A, and RAB3A in mouse Hb9 ESC-derived neuronal and glial cells
Ranieri et al., 2012 [51]	Rat	Hippocampal slices	20	N/A	9 d	200–250	Parallel to soma-dendritic axis of CA1 pyramidal cells	Recording of fEPSPs; recordings of gene induction	Anodal DCS up-, while cathodal DCS downregulates LTP induced by TBS; induction of early genes c-fos and Zif268 following neuronal activation
Chang, Lu, and Shyu, 2015 [20]	Mouse	Thalamocingulate slices	15	4	N/A	400	Parallel and perpendicular to direction of axodendritic fibers in the ACC	EPSCs in MEA and patch recordings	Cathodal DCS induces LTD via an NMDA-dependent mechanism
Sun et al., 2016 [18]	Mouse, Human	Coronal slices	10 or 25	8.18 or 10.18	1 d, 3 l	300 or 400	Parallel or orthogonal to the M1 fibers (L V to II/III projections)	Recording of fEPSPs, mGluR5-mTOR signaling as novel pathway in tDCS	Cathodal DCS induces LTD in both human and mouse cortex in vitro
Sun et al., 2020 [52]	Mouse, Human	Cortical slices	25	2.3	1 d, 3 l	400	Orthogonal to pia, parallel to vertical interlayer M1 projections; cathode proximal to cortical pia surface; anode beneath subcortical white matter	Recording of fEPSPs; immune-staining; KA-induced seizure model	DCS induced LTD-like plasticity in superficial cortical layers, and LTP-like plasticity in deep cortical layers; regional depression of cortical excitability is NMDA-dependent
Reato, Bikson, and Parra, 2015 [53]	Rat	Hippocampal slices	10	−20 to + 20	N/A	N/A	Parallel to CA3 pyramidal neurons	Changes in gamma power; MUA measurement	Induction of gamma oscillations by carbachol prior to DCS; altered gamma power and MUA after DCS; acute upregulation of MUA and power at positive fields; acute downregulation at negative fields
Zhao et al., 2015 [54]	Mouse	NPCs	90	115	1 d	0.25 nA	/	Migration assay	DC electric fields enhance cellular mobility; cell migration to the cathode via a calcium-dependent mechanism
